# A molecular overview of the primary dystroglycanopathies

**DOI:** 10.1111/jcmm.14218

**Published:** 2019-03-05

**Authors:** Andrea Brancaccio

**Affiliations:** ^1^ School of Biochemistry University of Bristol Bristol UK; ^2^ Istituto di Chimica del Riconoscimento Molecolare ‐ CNR c/o Università Cattolica del Sacro Cuore Roma Italy

**Keywords:** dystroglycan, dystroglycanopathies, missense mutations, molecular analysis, protein domains

## Abstract

Dystroglycan is a major non‐integrin adhesion complex that connects the cytoskeleton to the surrounding basement membranes, thus providing stability to skeletal muscle. In Vertebrates, hypoglycosylation of α‐dystroglycan has been strongly linked to muscular dystrophy phenotypes, some of which also show variable degrees of cognitive impairments, collectively termed dystroglycanopathies. Only a small number of mutations in the dystroglycan gene, leading to the so called primary dystroglycanopathies, has been described so far, as opposed to the ever‐growing number of identified secondary or tertiary dystroglycanopathies (caused by genetic abnormalities in glycosyltransferases or in enzymes involved in the synthesis of the carbohydrate building blocks). The few mutations found within the autonomous N‐terminal domain of α‐dystroglycan seem to destabilise it to different degrees, without influencing the overall folding and targeting of the dystroglycan complex. On the contrary other mutations, some located at the α/β interface of the dystroglycan complex, seem to be able to interfere with its maturation, thus compromising its stability and eventually leading to the intracellular engulfment and/or partial or even total degradation of the dystroglycan uncleaved precursor.

## 1 BACKGROUND

**Figure 1:**
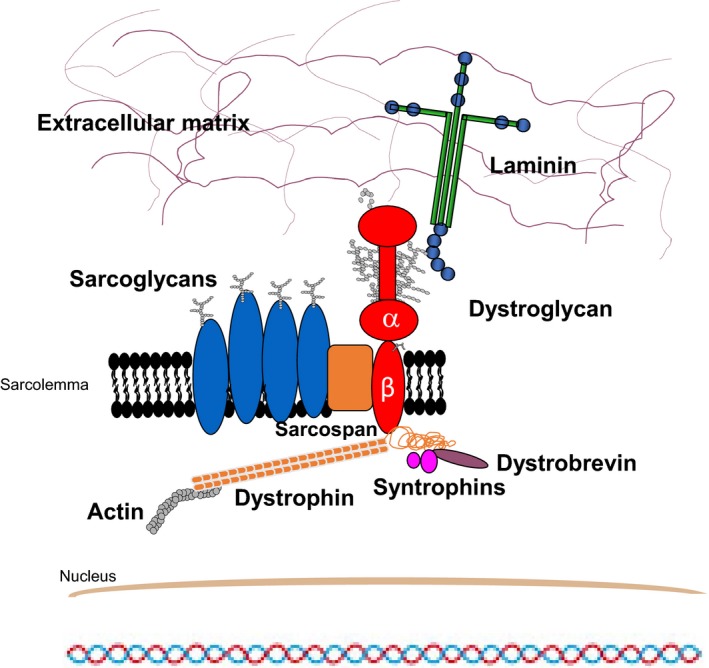
Schematic representation of the dystrophin‐glycoprotein complex (DGC) in skeletal muscle. The two dystroglycan subunits interact non‐covalently to form a bridge between the extracellular matrix and the actin cytoskeleton. α‐DG and β‐DG are non‐covalently connected and they also interact with numerous other proteins. The cytosolic domain of β‐DG is anchored to actin through the interaction with dystrophin and β‐DG also constitutes a scaffold for proteins involved in signal transduction such as Gbr2 and ERK. α‐DG is a so‐called peripheral membrane protein that interacts with the ectodomain of β‐DG on the extracellular side of the plasma membrane. α‐DG acts as a receptor for extracellular matrix proteins such as laminins (reported in the scheme), perlecan, neurexins and agrin among others

The dystroglycan (DG) adhesion complex takes center stage in a number of physiological and pathological contexts, playing a particularly important role in skeletal muscle.^1^ It is composed of two subunits, the extracellular and highly glycosylated α‐DG and the transmembrane β‐DG, which act as a molecular link forming an axis between the extracellular matrix and the internal cytoskeleton (Figure 1).^2^, ^3^ DG is the major non‐integrin cell adhesion complex and its role is to offer stability to a number of tissues: skeletal and smooth muscle, brain and peripheral nervous system, at the neuromuscular junction, at the interface between endothelial cells and the surrounding astrocytes end‐feet at the blood–brain barrier, in the kidney glomeruli basement membrane and in lungs at the epithelia‐connective border.^4^, ^5^, ^6^ In addition, DG is fundamental during mouse embryogenesis, which is blocked as early as day 6.5 in *dag1* knockout experiments.^7^

Interestingly, in invertebrates the full absence of DG, as in the *C elegans* knockout or a significant reduction in its expression levels, as in *Drosophila* do not give rise to muscle‐related phenotypes.^8^, ^9^ On the other hand, a family of patients in which some members displayed a homozygous frameshift mutation resulting in a complete lack of the DG complex, shows a condition leading to the severe Walker‐Warburg syndrome and brain‐associated problems (ie, tectocerebellar dysraphia), and to early lethality.^10^, ^11^

A few diverse α‐DG binding partners have been identified so far in different tissues, such as laminins, perlecan, agrin, neurexins, pikachurin and slits.^12^ All these proteins share laminin‐globular (LG) domains employed in binding extracellular α‐DG, and they require calcium to establish additional coordination contacts with the sugar moieties protruding from it.^13^ The DG affinity towards these ligands is generally high (K_d_s within the nanomolar range) and can be also influenced by the heterogeneous glycosylation of α‐DG.^12^

The α‐DG/laminin interaction is considered crucial for the stability of basement membranes. Intracellularly, the transmembrane β‐DG subunit does establish contacts with dystrophin and the cytoskeleton (see Figure 1). Due to these pivotal structural functions, DG and its associated proteins, as well as the enzymes responsible for its post‐translational maturation, are heavily involved in several forms of muscular dystrophy.^14^, ^15^ As a matter of fact sugar moieties, including a crucial phosphorylated O‐linked mannose,^17^, ^18^ that protrude from the central mucin‐like domain of α‐DG have been recently found to be important for efficient binding to matrix partners, and hypoglycosylation of α‐DG is thought to represent a distinctive molecular trait leading to several human pathologies, in particular to an increasing number of neuromuscular disorders.

## 2 THE EXPANDING GALAXY OF DYSTROGLYCANOPATHIES

Dystroglycanopathies are genetic diseases often arising from the hypoglycosylation of α‐DG and, depending on the affected genes they originate from, they are classified in the following main groups: (a) primary dystroglycanopathies, which occur when mutations of the *DAG1* gene alter the state of the DG core protein with potential repercussions on the glycosylation state of α‐DG; (b) secondary dystroglycanopathies, which depend on genetic abnormalities of *POMGnT1*, *POMT1* or *LARGE1* among others. These result in malfunctioning of the corresponding enzymes involved in the decoration with sugars of the DG core protein in the endoplasmic reticulum (ER) and Golgi, often affecting severely the glycosylation of α‐DG; (c) tertiary dystroglycanopathies, possibly involving genes (such as *ISPD* or *GMPPB*) and their corresponding enzymes responsible for the fabrication of the carbohydrate building blocks in the cytosol, thus indirectly modifying α‐DG glycosylation.^19^ The spectrum of secondary/tertiary dystroglycanopathies is likely to be even wider, since a link has been recently found between a dystrophic phenotype depending on α‐DG hypoglycosylation and mutations in protein complexes responsible for localizing proteins to the Golgi compartment.^20^ As opposed to the constantly growing number of secondary and tertiary dystroglycanopathies so far identified, only a few cases of primary dystroglycanopathies have been found in human patients as well as in zebrafish. In Table 1 is reported a re‐collection of the relevant pathologic and genetic details behind the mutations identified to date. Different phenotypes have been observed, ranging from mild muscular dystrophy with asymptomatic hyperCKemia to more severe limb‐girdle muscular dystrophy or Muscle‐Eye‐Brain disease.

**Table 1:** Mutations inducing primary dystroglycanopathies identified in vertebrates (human patients and zebrafish, *Danio rerio*) so far

Mutation	DG Subunit/Domain	Phenotype	Genotype	Reference
L86F^a^	N‐term/α‐DG (IG1)	Arrhythmogenic cardiomyopathy?	Heter.	21
V74I,D111N	N‐term/α‐DG (IG1)	Asymptomatic hyperCKemia, mild MD	Comp. heter.	22
T192M	N‐term/α‐DG (S6)	Limb‐girdle MD (LGMD2P), cognitive impair.	Homoz.	23
Stop codon at pos.266 (c.743delC ‐ single bp deletion)	N‐term/α‐DG (S6)	Severe WW‐phenotype, death upon birth	Homoz.	10
Stop codon at pos.398 (*D rerio*R398>stop, would correspond to R389 in humans)	mucin/α‐DG	Muscular Dystrophy	Homoz.	24
V567D (*D rerio*, would be I593 in humans)	C‐term/α‐DG (IG2)	Muscle degeneration, impaired mobility	Homoz.	25
C669F	N‐term/β‐DG (ectodomain)	Muscle‐Eye‐Brain,disease, multicystic leukodystrophy	Homoz.	26
R776C	β‐DG (cytodomain)	Late‐onset limb‐girdle muscular dystrophy	Homoz.	27

a It might represent a rare variant rather than a mutation. It is not clear if there is a contribution to the observed pathology coming from DG in this case. Moreover, no data have been collected to clarify whether α‐DG is abnormally glycosylated in this family.

## 3 HETEROGENEITY OF PRIMARY DYSTROGLYCANOPATHIES

**Figure 2:**
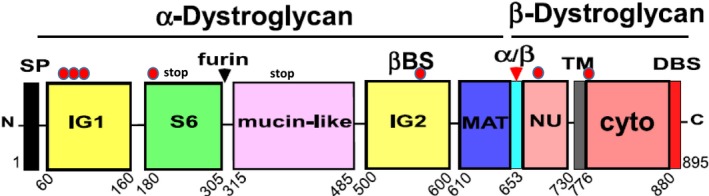
Domain structure of the dystroglycan complex with missense/nonsense mutations into their domain/structural context. Red dots at the top of the domain structure indicate the missense mutations so far identified. Namely, from left to right (N‐terminus to C‐terminus): V74I, L86F, D111N, T192M, V567D (zebrafish), C669F and R776C. A “stop” label marks the mutations introducing a frameshift reading error and a subsequent nonsense codon within the S6 domain (human) and the presence of a nonsense codon (in the mucin‐like region of zebrafish). SP: signal peptide, IG1 & IG2: immunoglobulin‐like domains, S6: domain similar to ribosomal protein S6, black arrow: furin cleavage site at Arg 312, mucin‐like: highly glycosylated and elongated central domain of α‐DG, MAT: the α/β maturation interface that includes the Gly‐Ser 653‐654 cleavage site (red arrow), βBS: β‐dystroglycan binding site on the IG2 domain, NU: natively unfolded – it refers to the ectodomain of β‐DG, TM: transmembrane stretch, Cyto: cytodomain of β‐DG which includes its dystrophin‐binding site (DBS)

Contrary to the increasing number of described secondary and tertiary dystroglycanopathies, primary dystroglycanopathies are comparatively less studied due to the small number of cases identified so far. *DAG1* mutations are rare, recessive mutations that are found in consanguineous families. No relevant effects of the *DAG1* mutations have been reported so far on their corresponding mRNA levels. The few mutations so far identified can be visualized in the context of the DG complex domain structure (Figure 2), as they have been found either within the N‐terminal region of α‐DG that represents an autonomous folding unit, or within regions located further downstream and involved in the formation and stability of the α/β DG complex.^1^, ^28^

Primary dystroglycanopathies arising from mutations at the N‐terminal region of α‐DG can present with a series of symptoms (ranging from severe to milder cases), in line with the variability of the phenotypes observed in secondary dystroglycanopathies. However, it is interesting to notice that in the cases analysed the DG complex does not suffer a major disruption, and although different degrees of hypoglycosylation can be observed (influencing the affinity towards laminin), the two DG subunits are produced and trafficked to the membrane. Thus, missense mutations within the N‐terminal region of α‐DG do not generally affect the overall folding, maturation and targeting of the DG complex to the plasma membrane. They are likely to act in a subtler fashion, ie by locally altering the structure of the first Ig‐like domain or of the S6 domain, or alternatively the overall flexibility of the whole N‐terminal domain. These effects would influence the “chaperoning role” that the N‐terminus of α‐DG exerts on the glycosyltransferase LARGE.^29^ Due to the observed high flexibility within the two subdomains of α‐DG N‐terminal region, it can be speculated that some of these mutations might also affect the interactions between DG and other enzymes important for the post‐translational glycosylation of DG in the Golgi (decoration process) still without inhibiting the maturation of the α/β complex into its two subunits.^30^, ^31^

On the other hand, both in human patients and zebrafish, phenotypes can also arise when mutations are found at the interface formed by α‐DG and β‐DG that is ultimately responsible for the non‐covalent interaction between the two subunits.^33^ In one case a missense mutation within the second Ig‐like domain of α‐DG, V567D, was shown to induce the *patchy‐tail* phenotype in zebrasfish typically caused by the total absence of DG.^25^ A complete lack of DG has also been observed in another zebrafish mutant in which a nonsense mutation was found within the mucin‐like region of α‐DG.^24^ It is worth to note that the case identified by Riemersma and colleagues (with a nonsense stop codon at the level of the S6 domain of α‐DG resulting in the full depletion of the whole DG complex) might represent the nearest human counterpart to these mutations.^10^

Our group has a long‐standing tradition of molecular studies on DG, for example by modelling and molecular dynamics, we have shown that the V567D zebrafish mutation, as well as its murine topological counterpart I591D, is likely to introduce a degree of instability/collapse within the α‐DG IG‐like β‐sandwich structure, leading to the exposure of some hydrophobic internal residues.^34^ In another case, the C669F mutation affecting the ectodomain of β‐DG was shown to cause a severe Muscle‐Eye‐Brain disease with a relevant phenotype involving the white matter in the brain displaying as multicystic leukodystrophy.^26^ Recently, we have shown that such conditions could depend on the intracellular engulfment within the ER of the DG unprocessed precursor, eventually leading to its likely ubiquitination and consequent degradation by the proteasome.^35^ It has yet to be assessed whether the pathologic consequences of this mutation depend on (a) the absence of DG properly targeted at the sarcolemma/plasma membrane, or on (b) the accumulation of intracellular DG due to its engulfment into the ER The evidence that no dominant negative effects have been observed in heterozygous carriers of the mutation seems to make the latter hypothesis less likely.^26^

It is perhaps not surprising that genetic abnormalities within the area responsible for the maturation of the DG complex, in which positions 653‐654 (Gly‐Ser in human) are highly conserved and represent the site for cleavage of the precursor into α‐ and β‐DG, result in very serious pathologies. Apparently in fact only a very small fraction of such an anomalous DG uncleaved precursor, whose partial degree of glycosylation is probably insufficient for efficient laminin binding, can make it to the membrane, where the correctly cleaved α/β–complex is not detected.^35^

The α/β interface of DG (ie C‐terminal domain of α‐DG and ectodomain of β‐DG), harbouring a SEA module, was shown to have an important role in post‐translational processing, trafficking and targeting of the entire complex.^36^ From an evolutionary standpoint, the α/β interface seems to be the most ancestral and the true Achille's heel of the complex, as revealed by experimental evidence of the whole α/β interface to constitute the target of some specific matrix metalloproteinases degrading action.^1^, ^37^ This may have important pathological consequences in tissue remodelling upon wound‐healing and chronic inflammation, leading to muscular dystrophy but also to cancer invasion.

Very recently a first mutation within the cytodomain of β‐DG, namely R776C, has been identified, causing a late‐onset form of limb‐girdle muscular dystrophy (see Table 1).^27^ This arginine is the first residue of the cytosolic domain of β‐DG, that is, it is part of its nuclear localization peptide and might represent a putative docking site for MAPK.^27^ Interestingly, the effect of R776C could also depend on it being a mutation in the basic sequence that governs membrane orientation of transmembrane proteins.^38^

## 4 CONCLUSIONS

New mutations in *DAG1* are likely to be identified in the future, and it will be interesting to assess their effect in view of the ongoing domain structural assessment and the possible collection of further additional structural information. A system and rationale for the classification of a larger amount of information (ie, mutations) based on the molecular structure of DG is likely to become a priority in the future, once a statistically significant amount of mutations has been characterised.

## CONFLICTS OF INTEREST

The Author declares that he has no competing interests.
